# Family Function, Loneliness, Emotion Regulation, and Hope in Secondary Vocational School Students: A Moderated Mediation Model

**DOI:** 10.3389/fpubh.2021.722276

**Published:** 2021-10-04

**Authors:** Pan Yun, Han Xiaohong, Yang Zhongping, Zhao Zhujun

**Affiliations:** ^1^School of Psychology, Guizhou Normal University, Guiyang, China; ^2^Guizhou Nursing Vocational College, Guiyang, China

**Keywords:** family function, loneliness, hope, emotion regulation, expressive suppression

## Abstract

This study explored family function as a key factor of loneliness, hope, and emotion related to secondary vocational school students during the novel coronavirus pandemic. Chinese versions of the Olson Family Function Scale, Russell Loneliness Scale, Snyder Hope Scale, and Gross Emotion Regulation Scale were completed by 5,138 participants. Guardian type significantly predicted family function and loneliness. Family function significantly and positively correlated with loneliness. The relationship between family function and loneliness was mediated by hope, and expressive suppression concurrently moderated the relationship between hope and loneliness. Our study offers meaningful insights into the family function of Chinese secondary vocational school students. The findings supported a moderated mediation model that exemplifies the relationship between family function, loneliness, expressive suppression, and hope. Although the results showed that high hope mediated lower family functioning and loneliness, low expressive suppression led to intense loneliness immediately. This study confirms that emotional strategy is important and associated with mental health. It also suggests that schools should pay more attention to students' emotion regulation and help them rebuild hope or appropriate cognition to relieve loneliness during crisis events.

## Introduction

A sudden life event can considerably affect adaptability and cohesion among members of a family. According to crisis theory, family members may become more or less cohesive in response to ongoing changes in the environment (1, 2). During the novel coronavirus 2019 (COVID-19) pandemic, every Chinese family faced unprecedented challenges in their daily life; this appeared to enhance family relationships in some, but not all, cases. Furthermore, tension, anxiety, and irritability were more likely to occur in senior students (3). Moreover, during the COVID-19 pandemic, Chinese citizens underwent home quarantine to prevent the spread of the virus; all of these phenomena indicate that family function during the COVID-19 pandemic is a topic worthy of consideration. Considering the reality of the Chinese society, secondary vocational school students remained at home longer than primary and high school students during the pandemic. This duration was based on the decisions made by the Chinese Ministry of Education. Traditionally, secondary vocational schools follow a boarding school model; however, during the pandemic, these students were required to stay at home, suggesting that these students may have faced greater challenges than other student groups in psychological state and external environment. Meanwhile, traditional Chinese education does not hold a generally positive view of secondary vocational school students; in the public's eye, they are often labeled as the “unsuccessful/underachieving group.” Accordingly, members of such groups tend to be affected by negative social identities that influence their cognition, attitudes, behaviors, and values (4). Moreover, a low social status typically affects subsequent employment status, thus forming a vicious circle that fosters lower self-expectations and self-efficacy (5). A study has also shown that secondary vocational school students exhibit relatively poor self-management and high internet addiction, and that 60% of these students do not foster interpersonal relationships (4). Therefore, in the present study we selected secondary vocational school students as our study sample.

## Theoretical Background and Hypotheses

### The Correlation of Family Function on Loneliness

Miller et al. (6) and Skinner et al. (7) defined functional families as those that provide the necessary materials for one's survival, and that have family members who provide mental health and/or sustainable social development support. Based on a Western academic viewpoint but in the Chinese context, researchers have found that family function should also include organization in the interactions among members and coordination in responding to external changes (8, 9). Furthermore, according to Olson's circumplex model (10, 11), family function is divided into three dimensions: cohesion, adaptability, and mutual communication.

The denotation of family as a dynamic system suggests that there may be mutual influence pathways among family members. Thus, adverse events could damage a family's dynamic balance (e.g., the death of family members, separation, role transitions, and changing connections between the social environment and family). When family relationships face such challenges, family members must acquire new rules and re-establish these relationships to solve various frictions, contradictions, and thereby maintain balance (12). Moreover, there were gender differences between family function and adolescents' emotional health, and gender acted a mediating role of their relationship (13, 14). Furthermore, research has shown that family function is influenced by whether the parents served as guardians; for example, single-parent households had more family conflicts due to increased behavioral problems of adolescents (15, 16). Therefore, both sex and guardians were considered as variables in this study.

The COVID-19 pandemic represents the most serious human public health challenge since the Spanish flu in 1918. Moreover, the psychological impact of this pandemic is experienced by the general public, such as patients, paramedics, college students, and the elderly (17). Accordingly, this social environment inevitably impacts the dynamic balance of family functions. In line with this view, a Chinese proverb says “seemingly in harmony, but actually at variance;” such a proverb is useful to understand the situation that this pandemic has brought upon families. People have been required to remain isolated at home for a long time during the pandemic, meaning that students have had more contact and opportunity than usual to communicate with their parents. However, although family members have been forced to stick together in the same locations, they may still be experiencing conflicts and loneliness. Particularly, undemocratic families (i.e., families that ignore children's personal preferences and opinions) could experience an aggravation in conflicts and problems. For example, Skinner's process model and McMaster's model of family functioning both assert that better family function leads to better mental health and physical status of family members (6, 7). A study by Kim and Baik (18) indicated that poor family function can increase loneliness levels in older adults. Another study reported that family function correlated with loneliness and that social support could play a mediating role in that relationship (19); moreover, Yang et al. (20) also found that family function was associated with loneliness in undergraduates. Therefore, it is necessary to explore the correlation between family function and loneliness to identify internal family-based factors related to mental health.

Although there are diverse definitions of loneliness, there are commonalities among the definitions. Several studies have indicated that loneliness is an emotional problem, a component of an individual's psychological or emotional experience, and a qualitative abnormality in the process of social interpersonal communication. Specifically, Weiss (21) defined loneliness as a subjective psychological feeling or experience. Perlman and Peplau (22) summarized loneliness as a painful, unpleasant, and negative emotion originating only from interpersonal relationships. Thus, loneliness is an unipolar emotion generated by the discrepancy between desired social communication and reality. It has been suggested that all lonely people understand and experience loneliness similarly (23). Studies have also shown that loneliness can be categorized into two components: (1) emotional loneliness represents emotional alienation that occurs in important, close relationships (e.g., among parents, lovers, or friends), and (2) social loneliness generally comprises emotional alienation from society, which is characterized by individuals being rejected by social groups or organizations (24).

The combination of Olson's circumplex model with this emotional loneliness dimension, leads us to infer that loneliness also occurs in situations where family function—specifically its cohesion dimension—is weak (10, 11). In fact, Deng and Zheng (25) indicated that family function significantly predicts emotional expressiveness, domestic affection, and social loneliness; in their study, undergraduates who scored higher on cohesion and adaptability were more likely to express positive emotions and less loneliness. Therefore, family function can predict the prosocial nature of interpersonal relationships, and a good family environment or better family cohesion can decrease the risk of loneliness (26). Hence, our first hypothesis is that there would be a significant correlation between family function and loneliness.

### Hope as a Mediator Between Family Function and Loneliness

Hope is a dynamic inner power and subjective experience that helps individuals to reshape their self-confidence and improve their capability, enabling them to pursue a better state (e.g., by addressing dilemmas); it encompasses psychology, physiology, and sociology, enabling individuals to establish positive beliefs, values, and engage in more pro-social behaviors, all of which allow people to overcome difficulties (27). The hope theory by Snyder et al. (28) suggests that the core components of hope are the cognitive mechanisms of pathway thinking and agency thinking. As a method for coping, hope can be useful for solving and buffering crises or pressures that individuals may face. Moreover, high levels of hope-related traits help maintain mental health (29). Subsequently, studies have found that depressed patients with greater dispositional hope gained positive psychological means to relieve stress-related emotions, facilitate physical rehabilitation, and re-integrate into society (30). Additionally, several medical studies have found that hope mediates the relationship between family function and quality of life (31). Gong (32) found that hope also acts as an intermediary between loneliness and quality of life. Further, Sharabi et al. (33) found that loneliness is closely related to hope and family, and that family cohesion is a direct predictor of hope levels. Thus, loneliness, hope, and family atmosphere are key factors for individual development; family atmosphere includes material and spiritual support (34), which facilitate the associations between family function, hope, and loneliness. Hence, our second hypothesis is that hope would mediate the relationship between family function and loneliness.

### Emotion Regulation as a Moderator

The core of emotional intelligence is emotional self-management, which involves emotion regulation, expression, motivation, reflection, and cognition (35). In this study, we focus on emotion regulation, which refers to individuals' control over their emotional responses as caused by physiological reactions, subjective experiences, or facial expressions (35). Gross and John's (36) emotional conditional process model proposed that different stages of event development generate different emotions; this model posited five different emotion regulation processes: episodic selection, modification, attention allocation, cognitive change, and response adjustment. In this model, individuals re-evaluate their input information and regulate their emotional response, finally outputting an emotional response that may, in itself, be suppressed or reappraised. For example, if an emotional experience is painful, people's cognition may rationalize the incident and try to minimize the generation of negative emotions (i.e., suppression), albeit it may also transform the character of the emotion and reaction (i.e., cognitive reappraisal).

According to the theory of Snyder (28) and Gross and John (36), hope as cognitive processing is connected with emotion regulation; for example, Peh's et al. (37) findings reported that hope shared a relationship with cognitive reappraisal, but not with suppression among patients with cancer. The construct correlation *(r* = 0.5) reported between the theoretical model of hope and the theory of optimism (38, 39), suggested that they were both positive components of cognitive processing. This means that people with higher hope should be able to sustain positive emotions to pursue goals with full energy and enthusiasm compared to people with lower hope. The higher hope individuals always had higher self-efficacy, meaning that they had enough power to achieve emotion regulation (40). Therefore, it is important to characterize the relationship between emotion regulation and hope.

Meanwhile, hope involves rational cognitive and behavioral tendencies acquired through learning, whereas loneliness is an undesired experience and emotion. During the COVID-19 pandemic, face-to-face social contact has been greatly restricted, and people have been facing challenges in their intimate family relationships. However, although adults have a rational cognitive “storybook” (i.e., hope), the output is not always a positive emotion because of the function of emotion regulation (28, 36). As previously shown, the two dimensions of emotion regulation (i.e., cognitive reappraisal and expressive suppression) have inconsistent relationships with psychological processes (41); accordingly, hope may separately link with cognitive reappraisal and expressive suppression (42). Previous studies showed that a negative event induces adolescents' lower hope level (43) and the higher hope individuals could achieve emotion regulation (40). As a result, people with different levels of hope may experience different emotions depending on the response to a life event: a cognitive reappraisal or an expressive suppression response. Thus, our third hypothesis proposes that cognitive reappraisal or expressive suppression moderates the correlation between hope and loneliness.

### This Research

Chinese secondary vocational school students are a special group in that they have the characteristics of adolescence but are also unique. The period of adolescence is characterized by rapid development of physical and mental mechanisms; Chinese secondary vocational school students are especially vulnerable to being despised by peers for having poor awareness, academic failure, and employment-seeking pressures (44, 45). Thus, from the perspectives of mental health and school management, Chinese secondary vocational school students may face more pressure than other student groups.

**Figure 1:**
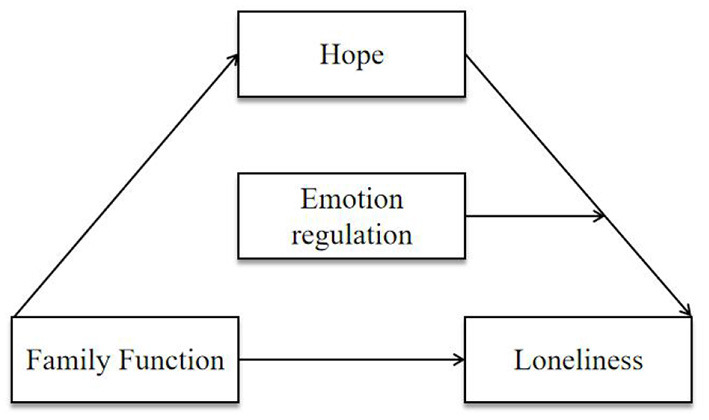
The proposed theoretical model between the interest variables.

Consistent with crisis theory, this research considered family function and emotion regulation as important predictors of loneliness. Both satisfied family function and healthy emotion regulation may prove protective during a time of crisis, such as the COVID-19 pandemic. Initially, we constructed a theoretical model shown in Figure 1; typically, family is an individuals' most important social support system, and a harmonious family atmosphere is conducive to reducing loneliness. Concomitantly, hope is an important predictor of individuals' physical and mental health and an important cognitive strategy that helps individuals adjust their mental health status during stressful situations (e.g., the COVID-19 pandemic). However, if hope is insufficient, individuals may still rely on emotion regulation, which may help reduce their psychological burden and negative feelings (e.g., loneliness). Therefore, individuals with a positive emotion regulation could effectively mobilize hope and loneliness to help them establish goals and even ease loneliness.

Thus, we propose the following hypotheses: (1) family function and loneliness have a significant positive correlation; (2) hope mediates the relationship between family function and loneliness; (3) emotion regulation (i.e., expressive suppression or cognitive reappraisal) moderates the relationship between hope and loneliness.

## Methods

### Participants

This study was conducted using a questionnaire survey among secondary vocational school students, in late April 2020. Secondary vocational school students underwent home, amidst the pandemic outbreak in China. Thus, teachers provided students with internet-based questionnaires; we collected data via a Chinese online crowdsourcing platform, similar to the Amazon Mechanical Turk. Convenience sampling methods were used to provide the questionnaire survey to those students, who participated voluntarily without reward. About 5,138 secondary vocational school students voluntarily completed survey (effective response ratio: 95.15%). Of these, 262 responses were invalid due to patterned or biased responses. Table 1 presents the demographic characteristics of the participants.

**Table 1:** Demographic characteristics (*N* = 5,138).

**Variables**	**Group**	***N***	**%**
Grade	Grade 1	2,920	56.83
	Grade 2	2,112	41.11
	Grade 3	106	2.06
Sex	Female	2,056	40.02
	Male	3,082	59.98
Guardians	Parents	4,311	83.90
	Non-parents	827	16.10

### Procedure

#### Family Function Questionnaire

The Chinese version of the Family Function Questionnaire has been used in prior research to assess family function in Chinese secondary vocational school students (10, 46). It comprises 30 items divided into the adaptability and cohesion dimensions; it showed high reliability and validity, with a Cronbach's alpha coefficient of 0.926, 0.935, and 0.963 and for the adaptability, cohesion, and overall scale, respectively. The cohesion dimension assesses individuals' emotional connection with family members, whereas the adaptability dimension refers to family members' decision-making and transformation when external factors change. Items of the questionnaire are rated on a 5-point Likert scale, ranging from 1 (*never*) to 5 (*always*), and assess actual and rational family responses; the greater the difference between actual and rational responses, the greater the degree of dissatisfaction.

#### Loneliness Questionnaire

The Chinese version of the loneliness questionnaire is a self-report measure that assesses satisfaction with the quantity and quality of social networking (23, 47). It comprises 20 items, each of which is rated on a 4-point Likert scale, ranging from 1 (*never*) to 4 (*always*), such that a higher score indicates greater loneliness. The Cronbach's alpha coefficient of the scale was 0.846.

#### Hope Questionnaire

The Chinese version of the hope questionnaire was used to measure cognitive and behavioral tendencies acquired through learning. During the process of achieving goals, individuals evaluate the internal and external situation to seek practical solutions through pathway and agency thinking (28). Pathway thinking is defined as the generation of a plan or methods for attaining goals, while agency thinking as directional energy or motivation, and both are components of hope (28). The 12 items of this scale are each rated on a 4-point Likert scale, ranging from 1 (*strongly disagree*) to 4 (*strongly agree*). The Cronbach's alpha coefficient for the overall scale was 0.845, while the values for pathway thinking and agency thinking were 0.775 and 0.777, respectively.

#### Emotion Regulation Questionnaire

The Chinese version of the emotion regulation questionnaire was used to measure the extent of cognitive reappraisal and expressive suppression (36, 48). Cognitive reappraisal is a positive and forward-thinking adjustment strategy used to re-evaluate situational emotional arousal, whereas expressive suppression is a negative behavior or emotional response strategy (41). It comprises 14 items, which are rated on a 7-point Likert scale, ranging from 1 (*strongly disagree*) to 7 (*strongly agree*). Moreover, the Cronbach's alpha coefficients for the overall emotion regulation and its two dimensions (cognitive reappraisal, expressive suppression) were 0.851, 0.877, and 0.801, respectively.

### Ethical Considerations

All students voluntarily participated in our survey, and they were informed that they could withdraw from the survey at any time. Additionally, each participants signed an electronic informed consent form before filling the survey questionnaire. Study procedures were approved as exempt by the morality and ethics committee of Guizhou Normal University for human subjects. Present study performed were in line with the principles of the 1964 Helsinki declaration (including its later amendments or comparable ethical standards).

### Data Analysis

SPSS 23.0 was used for data processing and analysis. First, invalid data due to patterned or biased responses were deleted after performing the frequency analysis. Second, common method biases were tested by performing factor analysis. Third, descriptive statistics and Pearson correlations were computed among for all the variables. Lastly, we employed Model 4 and 14 of the SPSS PROCESS macro (version 3.0) to examine the mediating role of hope and the moderating role of emotion regulation (including cognitive reappraisal and expressive suppression) in the relationship between hope and loneliness, respectively, based on the bias-corrected percentile bootstrap method [5,000 samples; (49)] with 95% confidence interval (CI), which was used for detecting significance effects. All data were standardized before analyses in Model 4 and 14, except for sex and main guardians, which were added as control variables in the models based on previous studies (50–52).

## Results

### Common-Method Bias Test

Due to the self-report nature of the data, there was a possibility of common method bias (53). Therefore, we used the Harman single factor test, as per Podsakoff et al. (54), to examine common method bias. Our results showed that the data were suitable for factor analysis (Kaiser-Meyer-Olkin coefficient = 0.95, *p* < 0.001). There were 17 factors with eigenvalues >1; the first factor explained 23.9% of the variance, which was lower than the criterion of 40%. Thus, there was no serious common-method bias in our study.

### Descriptive Statistics and Analysis

Table 2 presents the means, standard deviations, and *t* values for the study variables. Although male students generated higher values for emotion regulation than female students (i.e., Least significant difference test), the latter generated higher values for family function and loneliness (*p* < 0.05 for all values) than the first. When parents were the main guardian, secondary vocational school students had lower loneliness and family function scores, by final *post-hoc* test (*p* < 0.05).

**Table 2:** Means, standard deviations, and correlations among the study variables.

	**M ± SD**	**Sex**	**Guardian**	**Emotion regulation**	**Cognitive reappraisal**	**Expressive suppression**	**Hope**	**Loneliness**	**Family function**
Sex	1.40 ± 0.49	1							
Guardian	1.16 ± 0.37	0.029^*^	1						
Emotion regulation	4.49 ± 0.81	−0.161^**^	−0.007	1					
Cognitive reappraisal	4.98 ± 0.98	−0.095^**^	−0.030^*^	0.818^***^	1				
Expressive suppression	4.00 ± 0.99	−0.168^**^	0.019	0.821^***^	0.343^***^	1			
Hope	22.58 ± 5.35	−0.011	−0.015	0.138^***^	0.234^***^	−0.007	1		
Loneliness	42.61 ± 8.80	0.066^**^	0.045^**^	−0.001	−0.221^***^	0.217^***^	−0.249^***^	1	
Family function	10.96 ± 13.35	0.108^**^	0.061^**^	−0.009	−0.049^**^	0.034^***^	−0.043^**^	0.176^***^	1

* *p < 0.05*,

** *p < 0.01*,

*** *p < 0.001*,

*all two-tailed; the same notation is used in the subsequent tables*.

Family function was significantly positively correlated with loneliness (*r* = 0.176, *p* < 0.001)—supporting our first hypothesis. As Table 2 shows, family function was not associated with emotion regulation; however, the two dimensions of emotion regulation exhibited correlations in opposite directions with family function (Cognitive reappraisal: *r* = −0.049, *p* < 0.01; Expressive suppression: *r* = 0.034, *p* < 0.001).

Loneliness was significantly negatively correlated with hope (*r* = −0.249, *p* < 0.001), while hope was significantly negatively correlated with family function (*r* = −0.043, *p* < 0.01). Additionally, the expressive suppression dimension was positively correlated with loneliness (*r* = 0.217, *p* < 0.001) and family function (*r* = 0.034, *p* < 0.001), and the cognitive reappraisal dimension was positively correlated with hope (*r* = 0.234, *p* < 0.001). This suggests that the two dimensions of emotion regulation represent different, opposing mechanisms.

### Testing Hope as a Mediator

Following the determination of a significant interaction, we tested the second hypothesis. The mediation effect of hope on the relationship between loneliness and family function was examined using the SPSS Process macro Model 4 (49) after controlling for the demographic variables of sex and guardian (Table 3). Family function predicted loneliness (β = −0.042*, t* = −2.998, *p* < 0.01). The negative effects of family function on hope (β = 0.159*, t* = 11.832, *p* < 0.001) and of hope on loneliness were significant (β = −0.241, *t* = −18.127, *p* < 0.001). The indirect effect of hope on the relationship between family function and loneliness was significant [indirect effect = 0.01, Boot SE = 0.004, 95% CI = (0.003, 0.017)]. Despite the significant indirect effect, the direct effect between family function and loneliness remained significant [95% CI = (0.080, 0.114)], indicating that the mediation was only partial.

**Table 3:** Results of the mediation model.

**Variance**	**Model 1 Outcome variable: hope**			**Model 2 Outcome variable: loneliness**		
	**β**	**SE**	***t***	**β**	**SE**	***t***
Sex	−0.012	0.029	−0.433	0.092	0.027	3.386^***^
Guardian	0.039	0.073	0.536	0.084	0.036	2.309^*^
Family function	−0.042	0.014	−2.998^**^	0.159	0.013	11.832^***^
Hope				−0.241	0.013	−18.127^***^
*R^2^*	0.002			0.092		
*F*	3.564^*^			130.667^***^		

* *p < 0.05*,

** *p < 0.01*,

*** *p < 0.001*.

### Testing for Moderated Mediation

Following the determination of a significant interaction (Tables 2, 3), we examined the estimated conditional effects of hope on loneliness at different levels of expressive suppression by the Model 14 of PROCESS 3.0 macro for SPSS (55). Based on the above analyses, sex and guardian were chosen as control variables, in line with a previous study (52). Prior to testing the third hypothesis, we examined whether the effect of hope on loneliness was moderated by emotion regulation. The hope × emotion regulation interaction was not significant [*F*_(1,5,131)_ = 2.806, *p* = 0.094]; nor was the interaction between hope × cognitive reappraisal [*F*_(1,5,131)_ = 1.403, *p* = 0.236]. We examined the statistical significance of the moderated mediation effect using the bootstrapping method to generate CIs. This inferential test indicates that if the CI does not contain zero, then the indirect effect is moderated by the moderator variable (56). The analysis indicated that the moderating effect of cognitive reappraisal on the relationship between hope and loneliness was not significant [95% CI = (−0.001, 0.002)]. However, the hope × expressive suppression interaction was significant [*F*_(1,5,131)_ = 10.486, *p* = 0.001].

In line with a previous study (55), we tested four conditions: (1) effect of family function on hope; (2) effect of family function on loneliness; (3) interaction between hope and expressive suppression in predicting loneliness; and (4) different conditional level indirect effects of family function on loneliness, via hope, across low and high levels of expressive suppression. Table 4 presents the specifications of these models, where family function was negatively associated with hope [β = −0.042, SE = 0.014, *t*= −2.998, *p* < 0.001, 95% CI = (−0.070, −0.015)] and positively associated with loneliness [β = 0.147, SE = 0.013, *t* = 11.242, *p* < 0.001, 95% CI = (0.121, 0.172)]. Additionally, hope shared a negative relationship with loneliness (β = −0.238, *p* < 0.001), while expressive suppression shared a positive relationship with loneliness (β = 0.226, *p* < 0.001); since the interaction between hope and expressive suppression positively predicted loneliness [β = 0.029, SE = 0.012, *t* = 2.495, *p* < 0.05, 95% CI = (0.006, 0.051)], expressive suppression had a moderating effect on this relationship. These results supported the aforementioned first to third conditions.

**Table 4:** Results of the moderated mediation model analysis.

**Variance**	**Model 1 Outcome variable: hope**			**Model 2 Outcome variable: loneliness**		
	**β**	**SE**	***T***	**β**	**SE**	***t***
Sex	−0.012	0.029	−0.432	0.174	0.027	6.443^***^
Guardian	−0.032	0.038	−0.852	0.071	0.035	2.024^*^
Family function	−0.042	0.014	−2.998^**^	0.147	0.013	11.242^***^
Hope				−0.238	0.013	−18.356^***^
Expressive suppression				0.226	0.013	17.145^***^
Hope × expressive suppression				0.029	0.012	2.495^*^
*R^2^*	0.002			0.142		
*F*	3.555^*^			141.396^***^		

* *p < 0.05*,

** *p < 0.01*,

*** *p < 0.001*.

**Figure 2:**
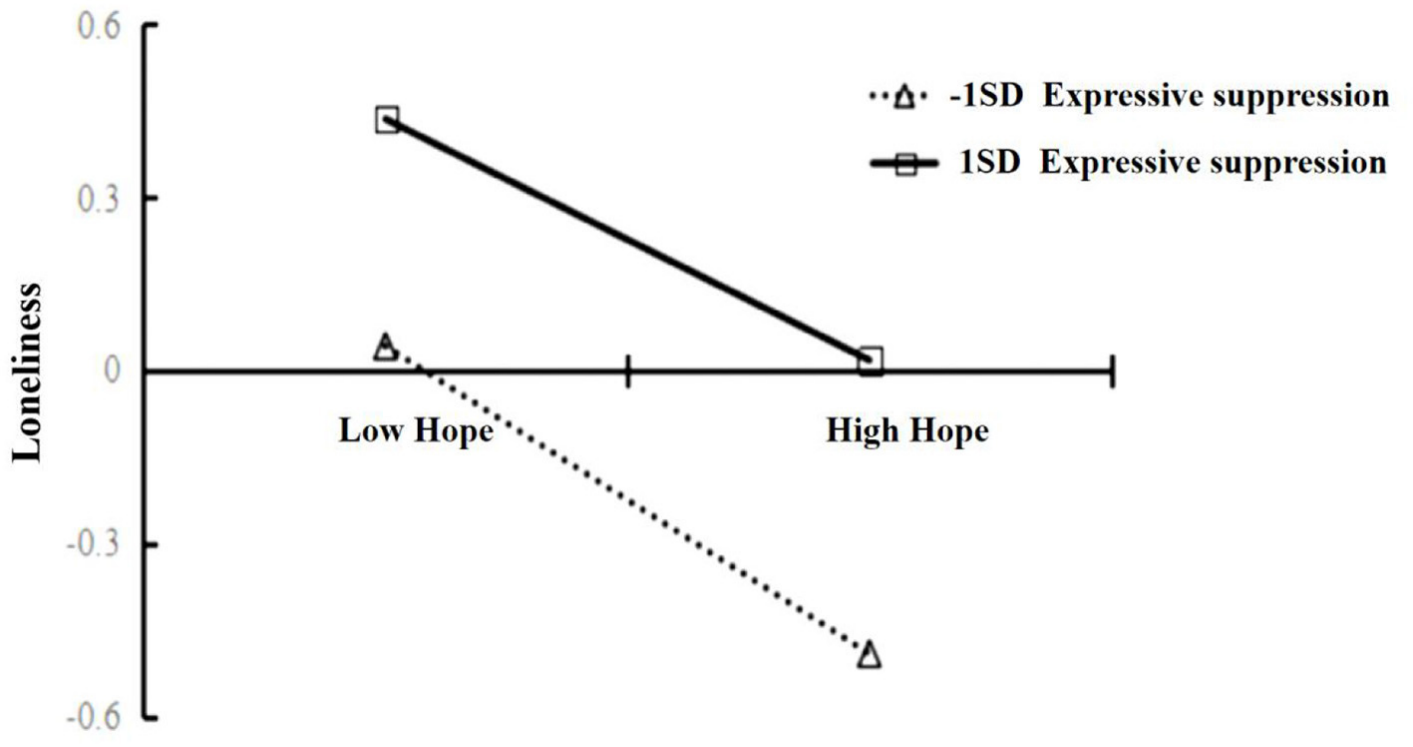
Predictions for the Interaction between loneliness and hope by expressive suppression values.

We also conducted a simple slopes test, while considering the interaction effect one standard deviation below and above the mean of the moderator. As shown in Figure 2, Table 5, the relationship between hope and loneliness was weaker when expressive suppression was lower [*b*_*slope*_ = −0.419, *p* < 0.001, 95% CI = (−0.244, −0.174); 1 SD above the mean] and stronger when expressive suppression was high [*b*_*slope*_ = −0.534, *p* < 0.001, 95% CI = (−0.300, −0.234); 1 SD below the mean]. Namely, higher expressive suppression and loneliness were experienced, when individuals experienced low hope. In summary, the interaction between family function and loneliness was mediated by hope, and the interaction between hope and loneliness was significantly moderated by the observed range of expressive suppression.

**Table 5:** Conditional indirect effects of expressive suppression on hope.

**Expressive suppression**	**Effect**	**SE**	***t***	**LLCI**	**ULCI**
M + SD	−0.210	0.018	−11.724^***^	−0.244	−0.174
M	−0.238	0.013	−18.356^***^	−0.263	−0.213
M – SD	−0.267	0.017	−15.817^***^	−0.300	−0.234

*LLCI, Lower Level Confidence Interval; ULCI, Upper Level Confidence Interval*;

*** *p < 0.001*.

## Discussion

The overarching intent of this study was to elucidate whether family function had close associations with loneliness, hope, and emotion regulation. Our results revealed that family function was positively correlated with loneliness, and that hope mediated the relationship between family function and loneliness. The results provided strong support for both hypotheses 1 and 2, and reported that expressive suppression could serve as a moderating role between hope and loneliness, that is, individuals with higher hope will have less loneliness than individuals with lower hope when they have higher expressive suppression—the latter moderation being one of the major findings of this study (hypothesis 3).

First, we explored whether parents or non-parents were the main guardians of secondary vocational school students affected loneliness and family function. As a result, we chose guardians as the control variable. However, even when parents were the main guardian, children whose parents tended to neglect them were more likely to report experiencing higher dissatisfaction with family function, psychological illnesses, and loneliness (57). Thus, children with neglectful parents were more likely to experience unpleasant emotions. Contrastingly, a cohesive, adaptable family usually fostered children's attachment and safety (58). Our results also indicated that male students' ability to regulate emotions were greater than that of female students; this was consistent with a previous study (59). Thus, sex was also chosen as a control variable. When female students experienced suppressive emotions, they were more likely to rely on their friends, perhaps sharing their feelings to gain social support. However, the COVID-19 restrictions meant that secondary vocational school students spent more time stay at home; without peer social support, women's emotion regulation may have been diminished and rumination may have been promoted, which led to increased attention to negative events and reduced motivation to release emotions (52).

Our results also showed a significant positive correlation between family function and loneliness, suggesting that greater dissatisfied family function can increase the risk of loneliness. Shek (60) previously reported that family function influences the healthy development of family members' minds and body, and that a good family environment could have a great impact on individuals' emotional responses and mental health. Thus, greater satisfaction with family function may be helpful for reducing loneliness, whereas dissatisfaction may intensify it. These results supported hypothesis 1. The basic unit in the ecological model of human development is family and peers (57), who may also be important for secondary vocational school students' development; studies before the COVID-19 pandemic indicated that loneliness promotes interpersonal anxiety (26). Additionally, the increased social isolation during the pandemic may have fostered loneliness to an even greater extent (61). Thus, based on Olson's circumplex model (11), our results highlight the critical role of family function during the pandemic; life uncertainties (e.g., those evoked by a pandemic) usually cause emotional and cognitive problems and damage mental health. Accordingly, we emphasize that further exploration of this topic is necessary.

Our results also reveal that hope mediates the relationship between family function and loneliness in secondary vocational school students, which is consistent with existing research (62). Thus, hope—as an optimistic state—can alleviate the emotions engendered by negative life events, which reveals a new perspective to explain the link between family function and loneliness in secondary vocational school students. Moreover, higher levels of hope could help maintain mental health and thereby decrease loneliness (29). Medical studies have found that hope mediates the relationship between family function and quality of life (31), and between social support and loneliness (63). Thus, dissatisfied family function could aggravate the experience of loneliness, but holding onto hope in these situations could encourage people to deal with family function and loneliness. These observations are consistent with our second hypothesis.

The most important finding of this study was the verification of hypothesis 3. Some studies have shown that emotion regulation is associated with loneliness (64, 65). However, in this study, only expressive suppression moderated the relationship between hope and loneliness. In line with this finding, our results also verify that individuals try to use emotion regulation strategies to suppress emotions before loneliness occurs. Expressive suppression played an important regulatory role in secondary vocational school students, as it positively correlated with loneliness; meanwhile, cognitive reappraisal was negatively correlated with loneliness. This indicates that the two types of emotion regulation have different underlying mechanisms (66). Although hope was negatively correlated with loneliness, this correlation transformed with expressive suppression; despite the relatively low beta coefficients, they were statistically significant, indicating that the relationship did exist. According to Gross's model (36), expressive suppression is an emotion regulation strategy, which is useful for emotional behavior control, but cannot mitigate physiological reactions or subjective emotional expressions. Expressive suppression has been consistently linked with avoidant attachment and estranged relationships. As our results showed that high hope individuals with lower expressive suppression were also intensely lonely. This indicates that emotional strategy is important and associated with situational flexibility; emotional strategy may help people regulate their states amidst challenging life events (36, 67, 68). Adolescents who adopt expressive suppression decrease negative emotions when they have a stronger social support system (69, 70). This suggests that lower family functioning could exacerbate students' situation, which may explain the important role of expressive suppression in our model. Moreover, we found that a sudden crisis event (i.e., the pandemic) led people to attempt to restrain their emotional intensity and output to maintain physiological and psychological balance. This finding is consistent with existing research on this topic (71). Contrastingly, Snyder (72) posited that hope is a belief of happiness; thus, hope is negatively related to loneliness. Nonetheless, hope levels fluctuated with people's emotions. Our findings showed that the predictive effect of hope on loneliness weakened with an increase in expressive suppression, indicating that higher levels of expressive suppression were closely related to loneliness in secondary vocational school students. Conversely, the predictive effect of hope on loneliness strengthened with lower levels of expressive suppression. Thus, expressive suppression regulated an individual's responsiveness to both loneliness and hope (42). In other words, secondary vocational school students with lower levels of expressive suppression can use hope to overcome loneliness.

Finally, we constructed a moderated mediator model, titled the “family function-hope-loneliness model;” expressive suppression regulated the relationship between hope and loneliness in this model. Most secondary vocational school students in this study were also limited by their age, which may denote a generally greater immaturity and relatively modest ability to self-differentiate. Therefore, because they had insufficient intrapsychic and interpersonal resources to cope with emotional experiences, the particular nature of the pandemic may have aggravated their loss of hope.

### Limitations

We collected the data during the COVID-19 pandemic; during this period, secondary vocational school students from diverse countries were differently excluded from school and educational management. Thus, future research is warranted to study the psychological status of different groups using comparative groups with a pre- and post-test design, and we aim to conduct such a study. This analysis would allow for the prediction of different psychological responses to similar crisis events, thereby, allowing for stakeholders to develop protective measures in advance.

Additionally, despite the relatively lower beta coefficients, especially in the interaction between hope and expressive suppression, the relationships between variables were statistically significant, indicating their existence, which may be due to the pandemic. Thus, subsequent research could focus on a longitudinal study to examine the stability of the model, considering the context of the current study.

Moreover, although the current sample was large, the sampling was regional, meaning it might not be representative of general vocational school populations; thus, cross-cultural studies are warranted to further explore behavior and cognitive differences regarding education or teaching methods. Additionally, this was a cross-sectional study at the family level, denoting the need for future studies to explore the influential factors of COVID-19 in schools and society more comprehensively.

Despite these limitations, we still believe that such analyses could help to stimulate or contribute to relative theory development, and even provide rationale for researchers who may invest time in longitudinal analyses that will test suggested processes more robustly. Our study findings make significant theoretical contributions, such as the role of hope as a mediator between family function and loneliness, and that of expressive suppression as a moderator between hope and loneliness. This study also has practical implications, as it is different from existing studies suggesting that emotion regulation affects loneliness (65, 73), and shows that expressive suppression plays an important role in loneliness in secondary vocational school students during the pandemic. Additionally, when these students showed lower levels of expressive suppression, the predictive effect of hope on loneliness strengthened significantly. Therefore, secondary vocational school students would likely benefit by seeking various strategies of suitable emotion regulation and incorporating hope or positive psychology to cope with life events.

### Conclusions

In conclusion, this study presented the role of family function in loneliness in secondary vocational school students. Hope was one pathway through which family function contributed toward reducing loneliness. Further, the effect of family function on loneliness was mediated by hope, which was thereby attenuated via expressive suppression. Our results highlight that family function was critical during the COVID-19 pandemic, as it had a strong relationship with loneliness. Although hope, as positive cognition, could reduce loneliness, expressive suppression decreased hope and increased loneliness.

## Data Availability Statement

The datasets presented in this study can be found in online repositories. The names of the repository/repositories and accession number(s) can be found in the article/supplementary material.

## Ethics Statement

The studies involving human participants were reviewed and approved by All procedures performed in studies involving human participants were in accordance with the Ethical Standards of Guizhou Normal University and with the 1964 Helsinki declaration and its later amendments or comparable Ethical Standards. Written informed consent to participate in this study was provided by the participants' legal guardian/next of kin.

## Author Contributions

PY designed and supervised the project. HX analyzed data and wrote up the original draft. YZ designed the structure and performed the calculations. ZZ discussed the results and proofread manuscript. All authors contributed to the article and approved the final manuscript.

## Funding

This work was supported by the Program for the Humanities and Social Sciences of Higher Education Institutions of Guizhou Province (Grant No. 2020WT012).

## Conflict of Interest

The authors declare that the research was conducted in the absence of any commercial or financial relationships that could be construed as a potential conflict of interest.

## Publisher's Note

All claims expressed in this article are solely those of the authors and do not necessarily represent those of their affiliated organizations, or those of the publisher, the editors and the reviewers. Any product that may be evaluated in this article, or claim that may be made by its manufacturer, is not guaranteed or endorsed by the publisher.

## Abbreviations

CI: confidence interval
COVID-19: novel coronavirus 2019
LSD: least significant difference
SD: standard deviation.
